# Addressing sexual violence as a weapon of war: A critical call for action in eastern Democratic Republic of Congo

**DOI:** 10.1002/ijgo.70446

**Published:** 2025-08-13

**Authors:** Aymar Akilimali, Fabien Balagizi, Priyadarshini Bhattacharjee, Nathan Mugenyi, Amos Kipkorir Langat, Adolphe Karegeya, Innocent Mufungizi, Gaurang Narayan, Dieudonné Kakusu, Freddy Zihindula, Isaac Isiko, Styves Banga, Lucien Baraka, Denis Mukwege, Olivier Nyakio

**Affiliations:** ^1^ Department of Research Medical Research Circle (MedReC) Goma Democratic Republic of Congo; ^2^ Society for Maternal‐Fetal Medicine (SMFM) Washington District of Columbia USA; ^3^ Translational and Clinical Research Institute Faculty of Medical Sciences, Newcastle University United Kingdom; ^4^ Department of Gynecology and Obstetics, Faculty of Medicine Mbarara University of Science and Technology Mbarara Uganda; ^5^ Department of Pharmacy, Faculty of Health Sciences Victoria University Kampala Uganda; ^6^ Pan African University for Basic Sciences Technology and Innovation Nairobi Kenya; ^7^ College of Nursing University of Utah Salt Lake City Utah USA; ^8^ Department of Obstetrics and Gynecology Indira Gandhi Government Medical College and Hospital Nagpur Maharashtra India; ^9^ Faculty of Medicine Evangelic University in Africa Democratic Republic of Congo; ^10^ Department of Community Medicine, Axel Pries Institute of Public Health and Biomedical Sciences Nims University Jaipur Rajasthan India; ^11^ Department of Gynecology and Obstetics, Faculty of Medicine Official University of Bukavu Democratic Republic of Congo

**Keywords:** Democratic Republic of Congo, sexual violence, survivor‐centered care, trauma‐informed interventions, weapon of war

**Abbreviations**
DR Congo: Democratic Republic of the Congo
HEAL Africa: Health, Education, Action, Leadership (a Congolese NGO)
LGBTQ+: Lesbian, Gay, Bisexual, Transgender, Queer/Questioning, and others
M23: March 23 Movement (a rebel group in DRC)
MONUSCO: United Nations Stabilization Mission in the Democratic Republic of the Congo
NGOs: Non‐Governmental Organizations
PTSD: Post‐Traumatic Stress Disorder
SMFM: Society for Maternal‐Fetal Medicine
SOFEPADI: Solidarité des Femmes pour le Développement Intégral (a Congolese women's rights organization)

The eastern Democratic Republic of Congo (DR Congo) is an ongoing conflict zone that experiences organized sexual violence tactics that both serve to intimidate local populations and defend occupied territories.^1^ DR Congo experienced ramped‐up military confrontation between several government forces and the M23 rebel unit and over 120 rebel groups that caused the displacement of 1.7 million civilians and intensified humanitarian disasters in 2023.^2^ Since its evacuation in 2023, the United Nations Stabilization Mission in the DR Congo (MONUSCO) has fallen short of sustaining mobile crime courts alongside evidence collection tasks, thus creating space for legal considerations.^3^ Conflicts in this context have transformed into a symbol of sexual violence, including rape as well as sexual slavery and forced pregnancy. This experience remains an obstacle for survivors trying to access justice and care.^4^ This paper investigates the core causes behind violence while assessing current intervention methods and offering practical solutions for healthcare practitioners and policy developers to control this human rights emergency.

Sexual violence continues to be a problem in the eastern DR Congo because of colonial exploitation that led to the development of both exploitative economic systems and racial conflicts. Following independence dictatorships together with genocide spillover effects contributed toward an increase in armed groups that extracted natural resources, including coltan and gold for survival. Analysis conducted by Solidarité des Femmes pour le Développement Intégral (SOFEPADI) shows that reported sexual violence cases involving women and girls stand at 78%, but cases involving males have increased to 22% since 2020, amounting to a double‐figure surge. Militarized masculinity and targeting practices directed at lesbian, gay, bisexual, transgender, and queer or questioning individuals and ethnic minorities among other groups have increased in both scale and scope according to researchers.^4^, ^5^

Years of official statistics show that when sexual violence cases reach law enforcement, there is just 12% investigation success followed by only 2% conviction rates.^5^

Cultural standards enhance the process through which survivors become marginalized by society. The patriarchal system chooses to punish victims by labelling them as family dishonorers, which causes both isolation and forced marriages to their attackers.^5^ According to HEAL Africa research in Masisi Territory rural areas in 2023, interviews established that 34% of survivors were rejected by family members when they reported sexual assaults.^1^, ^3^

Through two major legislative changes, the Congolese Government passed both the *Sexual Violence Act* of 2006 and established the Tribunaux Militaires de Garnison (Military Garrison Courts) in 2018. The departure of MONUSCO soldiers has led to more instability throughout the affected area. MONUSCO supported military mobile courts that convicted 148 offenders of sexual violence crimes from 2017 to 2022.^6^ The troop withdrawal created an obstacle for 63 ongoing cases and forensic training delays that made it harder for survivors to receive justice. Various legal reforms face obstacles because of inadequate funding alongside corruption within the system. During the 2022 audit, inspection officials discovered that 45% of mobile court funding had disappeared through corruption, thus delaying trials and with accompanying delays to protection schemes.^2^, ^3^

Non‐governmental organizations successfully occupy the essential roles that public institutions have failed to fulfill. Dr. Denis Mukwege established Panzi Hospital as a facility that combines medical services with legal assistance and social rehabilitation programs for patients. Panzi Hospital assisted 8200 survivors during 2020–2023 and managed to obtain 72% of convictions through their mobile court partnerships.^4^, ^7^ Through its HEAL Africa community advocacy networks, the organization has established 15 advocacy networks in North Kivu, which resulted in a 30% increase in reporting activity across those communities.^5^, ^8^

There is an immediate need for stable funding sources to replace terminated foreign assistance projects, such as those of the European Union and Canada. Donor funding needs to be shifted to establish mobile healthcare services and legal support systems. In 2019, the Kavumu model was demonstrated to be successful as clinicians, police and lawyers teamed up to bring 11 militia members to justice through their integrated services.^9^ Duplicating this method requires money for safe evidence management with protection for witnesses and outreach services for communities.

The International Criminal Court needs to intensify its investigation of sexual violence cases as part of crimes against humanity in the DR Congo, while third‐party states should implement universal jurisdiction laws to prosecute perpetrators who live outside the country. The government of DR Congo must establish independent oversight. The eastern DR Congo requires urgent joint intervention measures that target impunity systems while placing victim needs first. The healthcare community together with funding supporters and global health organizations need to celebrate local achievements demonstrated at Panzi Hospital and encourage continued financial backing as well as legislative change. Continued attention on this region will help end the pattern of violence and establish healing alongside justice in this highly traumatized area.

## AUTHOR CONTRIBUTIONS

Conceptualization: Aymar Akilimali, Priyadarshini Bhattacharjee and Fabien BALAGIZI, Investigation: Amos Kipkorir Langat and Styves Banga, Project administration: Olivier Nyakio and Aymar Akilimali, Resources: Nathan Mugenyi, Supervision: Olivier Nyakio, Priyadarshini Bhattacharjee, and Denis Mukwege, Validation: Adolphe Karegeya, Dieudonné Kakusu and Priyadarshini Bhattacharjee, Visualization: Lucien BARAKA, Aymar Akilimali, Isaac Isiko and Freddy Zihindula, Writing—original draft: All Authors, Writing—review & editing: All Authors, Final approval of manuscript: All Authors.

## FUNDING INFORMATION

The authors did not receive any financial support for this work. No funding has been received for the conduct of this study.

## CONFLICT OF INTEREST STATEMENT

All authors declare that they have no known competing financial interests or personal relationships that could have appeared to influence the work reported in this paper.

## Data Availability

Data sharing is not applicable to this article as no new data were created or analyzed in this study.
